# Supplementary material for: Are carbon emissions trading and green financial instruments synergistic? -Comprehensive quantitative research based on content analysis
Source: PLoS One. 2024 Mar 7;19(3):e0298601. doi: 10.1371/journal.pone.0298601 (PMC10919601; doi:10.1371/journal.pone.0298601)
Supplement: S1 Data — (ZIP) [file pone.0298601.s001.zip › Data/地级市Z_F工作报告.zip!/╒π╜¡/╝╬╨╦╩╨/└┤╫╘╣1⁄2╓┌║┼í╛┬φ┐╦╩2╛▌═°í┐_2018.docx]

各位代表：

　　现在，我代表市人民政府向大会报告工作，请予审议，并请市政协委员和其他列席人员提出意见。

　　2017年工作回顾

　　2017年是本届政府任期的第一年。市政府以迎接党的十九大召开、学习贯彻党的十九大精神为强大动力，在中共嘉兴市委的正确领导下，全面落实中央和省委、省政府的决策部署，统筹抓好稳增长、促转型、抓改革、强创新、治环境、惠民生、防风险等各项工作，较好地完成了市八届人大一次会议确定的目标任务。

　　——经济实力再跃新高。全市地区生产总值突破4000亿元，达到4355.2亿元，增长7.8%，人均GDP达到13917美元。财政总收入增长14.2%，增速居全省第一，其中市本级增长18.2%；实现一般公共预算收入443.8亿元，增长13.2%，总量跃居全省第四，其中市本级143.1亿元，增长16.1%；税收收入占一般公共预算收入比重达到92.8%，居全省第一。固定资产投资突破3000亿元；进出口总额、社会消费品零售总额分别增长19.4%、10.3%。经济运行呈现优化平稳、稳中向好、好于预期的发展态势。

　　——发展活力持续增强。大众创业、万众创新蓬勃开展。全市各类市场主体净增9.3万户，其中企业主体增加2.2万户。新增上市公司9家，累计达到53家。全社会研究与试验发展经费支出占地区生产总值比重达到3%，居全省第二；发明专利授权量增长11.9%；秀洲国家高新区发展排名列全国新晋国家级高新区首位，嘉兴科技城获批国家双创示范基地。新产业、新业态、新模式不断涌现，规上信息制造业产值达到1178亿元，增长14.9%，网络零售额达到1454亿元，增长30.6%。快速崛起的新动能正在深刻重塑全社会生产生活方式。

　　——城市影响显著扩大。连续四年成功承办世界互联网大会·乌镇峰会，连续三届荣膺“全国文明城市”称号，喜获全国社会治安综合治理最高荣誉“长安杯”，城市知名度和美誉度进一步提升。牢筑接轨上海“桥头堡”，召开推进省全面接轨上海示范区建设大会，签订合作协议30项，引进格科微电子等上海亿元以上项目52个、高层次人才319名，嘉兴在长三角区域中的重要地位更加凸显。

　　党的十九大闭幕仅一周，习近平总书记带领新一届中央政治局常委集体瞻仰南湖红船，参观南湖革命纪念馆，发表重要讲话。嘉兴作为党的诞生地和“红船精神”发源地，地位和影响力得到了空前提高，全市上下深受鼓舞、倍感振奋。

　　——城乡风貌加快改善。作为国家城市总体规划编制试点地区，高标准启动《嘉兴市城市总体规划》修编。城市有机更新步伐加快，全市拆除各类违法建筑2151万平方米，整治改造老旧住宅小区74.8万平方米；市区开工建设南湖湖滨公园、子城遗址公园，东栅老街片区更新改造工作稳步推进，打通三环南路、三元路（东升路-城北路段）等断头路，建成海绵城市试点项目102个。29个镇（街道）通过省小城镇环境综合整治考核验收，其中乌镇镇、姚庄镇位居全省前两名。美丽乡村建设全面升级，新增美丽乡村示范镇10个、特色精品村50个、3A级景区村庄15个。

　　——民生福祉不断增进。财政民生支出增长11.3%，占一般公共预算支出比重保持在80%。健全“大救助”体系，落实支出型贫困家庭救助政策，发放各类救助金2.6亿元。全市最低工资标准提高到1800元，最低生活保障标准提高到796元。城镇居民人均可支配收入突破5万元大关，达到53057元，增长8.4%，其中市本级增长8.8%；农村居民人均可支配收入达到31436元，增长8.4%，其中市本级增长8.6%；城乡收入差距比1.69︰1，继续保持全省最低。农村居民人均可支配收入连续十四年居全省第一。

　　一年来，嘉兴的变化是全方位、深层次、广领域的，取得的进步来之不易，赢得的成绩令人振奋，愈发坚定了我们沿着选定的方向大踏步前进的信心和意志。山高人为峰，路远脚更长。只要我们志存高远、脚踏实地，不动摇、不懈怠、不折腾，嘉兴迎来的定是更为辉煌灿烂的明天。

　　一年中，我们重点抓了以下六个方面：

　　（一）坚持供需两端发力，经济结构加快调整

　　以供给侧结构性改革为主线，聚焦实体经济主攻方向，注重发挥有效投资的关键性作用。

　　扎实推进“三去一降一补”。深入实施“退低进高”“退散进集”行动，淘汰1318家企业落后产能，处置“僵尸企业”30家。落实房地产调控政策，规范房地产市场秩序，商品住宅去化周期保持在合理区间。启动长三角最优金融生态区建设，加强企业“两链”风险排查和处置，全市金融机构不良贷款率0.64%，保持全省最低。落实“降本减负”系列政策，累计减免企业税费191.5亿元。继续突出重点，补齐短板、加强弱项。

　　大力提振实体经济。坚持制造业强市不动摇，联动推进“四换三名”“四加三减”“三强一制造”。完成工业技改投资1086.2亿元，新增工业机器人1848台、浙江名牌23个；全市制造业贷款余额比年初增加76.7亿元，增量居全省第一；规上工业增加值达到1727.3亿元，居全省第三，其中高新技术产业、战略性新兴产业占比分别达到47.1%和36%，均居全省第二。建筑业产值突破1000亿元。出台推进军民融合产业发展政策意见，省级军民融合示范基地达到3家。大力发展现代物流、科技与信息、金融、文化旅游、健康服务等现代服务业，旅游总收入首次突破1000亿元，税收亿元楼宇增加到16幢，服务业增加值达到1911.3亿元。深化农业供给侧结构性改革，实施农业转型发展三年行动计划，新增粮食生产功能区7.9万亩、绿色农产品基地2.1万亩，第一产业增加值达到134.7亿元。

　　积极扩大有效投资。实施“重点平台千亿投资、招商落地千亿投资”工程，市域外配水工程启动建设，钱江通道北接线二期、扩大杭嘉湖南排等重大基础设施项目加快推进，宝沃汽车、恒天祥符荡文创产业园等总投资超50亿元的重大产业项目集中开工。全市固定资产投资增长11.2%，其中重大基础设施、重大产业、高新技术产业和生态环保投资分别增长38.9%、21.8%、23.6%和61.8%。民间投资增长23.4%，增速居全省第一，占固定资产投资比重达到66.4%。

　　（二）坚持创新引领发展，“双创”生态持续优化

　　以“两区一城一走廊”建设为核心，统筹推进创新体制改革、创新主体发展、创新要素集聚、创新文化培育。制定人才新政三十条、科技新政二十条，突出奖补到人、激励到团队，启动16项省全面创新改革试验区重大改革项目。深化省校合作，注重战略谋划，加快超前布局，设立浙江清华柔性电子技术研究院、浙江未来技术研究院等，互联网+VR/AR、人工智能、未来生命、动态感知、量子通信等一批以数字经济为代表的战略性新兴产业落地生根。深入推进新型智慧城市标杆市建设，城市大数据中心和城市综合运营管理中心初具规模。积极打造以“全域孵化器”为特征的在长三角具有特殊影响力的科技企业孵化之城，新增各类孵化机构31家、省级企业研究院27家、高新技术企业241家、科技型中小企业637家。秀洲光伏小镇、嘉善归谷智造小镇、海宁阳光科技小镇被列为省级高新技术特色小镇。与杭州、松江签订《沪嘉杭G60科创走廊建设战略合作协议》，共同发布“嘉兴宣言”，科创走廊建设上升为省级战略。连续八年实施“创新嘉兴·精英引领计划”，连续九年举办“星耀南湖·精英峰会”，建成院士专家工作站73家，其中全国示范院士专家工作站3家；新增“国千”人才54名，累计达到215名。

　　（三）坚持全面深化改革，发展活力不断增强

　　以“最多跑一次”改革为牵引，撬动各领域改革取得积极成效。全面实施“一窗受理、集成服务”，在全省率先上线“一体化投资项目在线审批监管平台2.0版”，行业许可“一证化”改革全国首创，“最多跑一次”实现率达到94.6%。扎实推进资源要素差别化配置改革，开展新一轮工业企业综合绩效评价及结果应用，“区域能评、环评+区块能耗、环境标准”改革实现县（市、区）全覆盖。财税、国资改革取得新成效，全市政府产业基金规模达到93亿元，国有企业利润增长103%。深化农村产权制度改革，在696个行政村开展土地确权登记颁证工作，市、县、镇三级农合联组织运行机制进一步完善,提前全面消除年经常性收入低于50万元的经济薄弱村。统筹推进国家新型城镇化综合试点、国家医养结合试点、国家排污许可制度改革等61项国家、省改革试点，入选联合国国际可持续发展试点城市，嘉善县域科学发展示范点第一阶段建设圆满收官，新一轮发展改革方案获得中央和国务院批复。

　　（四）坚持对内对外开放，开放步伐明显加快

　　接轨上海全面提速提质。制定出台省全面接轨上海示范区创建三年行动计划，示范区建设全面发力、多点突破。编制完成沪嘉轨道交通接轨规划方案；选派115名干部赴上海挂职；引进上海科技创新载体18家，签订科技合作项目154项，在上海启用全国首张跨省域科技创新券；张江长三角科技城平湖园、上海自贸区（嘉善）协作区等毗邻区域合作平台集聚效应显现，全市省级以上开发区与上海各类开放创新平台合作实现县（市、区）全覆盖。滨海开发开放取得新成效，临港现代装备·航空航天产业园初具规模，嘉兴港全年完成集装箱吞吐量144万标箱、货物吞吐量8829万吨，规模分列全省第二和第三位。坚持“引进来”和“走出去”，启动高质量外资集聚地建设，引进世界500强、全球行业领先企业和总投资超亿美元项目40个，实际利用外资30亿美元，浙商回归省外到位资金645亿元；完成境外投资12.7亿美元，其中“一带一路”沿线国家投资8.2亿美元。继续做好对口支援、对口合作、山海协作等工作。

　　（五）坚持绿色协调发展，生态环境显著提升

　　以中央环保督察为契机，加大突出环境问题整治力度，持续推进“三五共治”。开展劣V类水剿灭行动，全市1341个劣V类小微水体完成整治，市控以上劣V类水质断面实现“清零”，Ⅳ类以上水质断面比重提高到97.3%，Ⅲ类以上达到38.4%。严格推行烟花爆竹“双禁”，完成13家热电企业超低排放改造，淘汰燃煤小锅炉650台，新增光伏并网容量758兆瓦，累计达到2049兆瓦，占全省三分之一。市区PM2.5平均浓度下降4.5%。有针对性制定实施“五废共治”三年行动计划，全面开展城乡生活垃圾分类处理，建成固体废弃物处置设施7个。扎实推进“绿城花海”建设，全市新建绿道36.6公里，实现省级森林城市县（市、区）全覆盖。

　　（六）坚持以人为本理念，人民生活提质改善

　　统筹就业创业政策，提供全方位公共就业服务，新增城镇就业11.5万人。推进全民参保计划，新增基本养老保险参保人数23万人、基本医疗保险参保人数8万人，实现全市医保实时结算和跨省异地就医（住院）结算。大力发展教育事业，中小学课程改革持续深化，首次新高考平稳实施，教育基本现代化县（市、区）创建取得新进展。高水平建设“健康嘉兴”，在全省率先建立长期护理保险制度，“双下沉、两提升”“分级诊疗”等工作有序推进；医疗服务接轨上海驶上“快车道”，全市公立医院与上海32家三甲医院建立紧密型合作关系，开设专家专病门诊150个，诊疗人次达5.6万，完成高难度手术3000多台，努力实现嘉兴百姓在家门口就能享受上海高端优质医疗服务。实施国家公共文化服务体系示范区后续建设行动计划，开工建设马家浜文化博物馆，建成市博物馆二期，继续办好端午民俗文化节、市民文化节、乌镇戏剧节等活动。举办第五届市民运动会、首届体育产业博览会，嘉兴籍运动员在第十三届全运会上取得历史最佳成绩。

　　平安嘉兴、法治嘉兴建设不断提升。“自治、法治、德治”基层治理工作创新写入十九大报告，全面建成镇（街道）综治工作、市场监管、综合执法、便民服务“四个平台”。深入开展安全生产、消防安全大排查大整治行动，安全生产事故起数、死亡人数分别下降35.6%和22.8%。积极预防和化解社会矛盾纠纷，加强社会治安综合治理，严厉打击各类违法犯罪活动，实现省级平安市、县（市、区）创建“十三连冠”“满堂红”。国防建设、双拥优抚、人民防空等工作进一步加强，民族宗教、档案史志、外事侨务、工青妇、科协、文联、残疾人、慈善、红十字等各项工作取得新成效。市人代会确定的十件民生实事项目目标任务如期全面完成，群众得到了更多实惠。

　　各位代表，过去一年，我们稳中求进、真抓实干，勠力同心、攻坚克难，在决胜全面建成小康社会进程中迈出坚实步伐。这些成绩的取得，是全市上下凝心聚力、拼搏奉献的结果。在这里，我代表市政府，向全市人民，向人大代表、政协委员和离退休老同志，向各民主党派、工商联、无党派人士、各人民团体和社会各界人士，向驻嘉人民解放军、武警官兵，向关心支持嘉兴发展的海内外朋友，致以衷心的感谢和崇高的敬意！

　　前进的道路从不会一帆风顺。我们也清醒认识到，面对人民日益增长的美好生活需要，发展中不平衡不充分问题仍然突出。创新发展的能力还不够强，传统产业改造提升不够快，新旧动能接续转换的任务依然艰巨；资源环境约束紧张，治水治气治废需要进一步加大力度；中心城市集聚辐射作用有待增强，城市功能品质还需提升；民生领域尚有不少短板。此外，少数政府工作人员服务意识不强，一些领域腐败问题时有发生。市政府将高度重视这些问题，采取更强有力的措施加以解决。

　　2018年工作任务

　　新的一年孕育新的希望，新的起点昭示新的辉煌。2018年是贯彻党的十九大精神的开局之年，是改革开放40周年，也是决胜全面建成小康社会、实施“十三五”规划承上启下的关键一年。嘉兴作为党的诞生地和“红船精神”发源地，必须把深入学习贯彻党的十九大精神作为当前和今后一个时期的工作主题，结合时代特点大力弘扬“红船精神”，不忘初心、牢记使命、永远奋斗，全力打造践行习近平新时代中国特色社会主义思想的示范地。

　　今年政府工作的总体要求是：高举习近平新时代中国特色社会主义思想伟大旗帜，全面贯彻党的十九大和十九届二中、三中全会精神，贯彻落实全国“两会”精神，坚持稳中求进工作总基调，坚持新发展理念，紧扣社会主要矛盾变化，按照高质量发展的要求，统筹推进“五位一体”总体布局和协调推进“四个全面”战略布局，坚定不移沿着“八八战略”指引的路子走下去，以供给侧结构性改革为主线，深入推进经济转型、城市转型、社会转型，全面抓好改革开放、产业升级、改善民生、整治环境、防范风险各项工作，加快建设具有国际化品质的现代化网络型田园城市，为打造富强民主文明和谐美丽的社会主义现代化强市奠定坚实基础。

　　综合考虑各方面情况，建议2018年全市经济社会发展的主要预期目标为：地区生产总值增长7.5%左右，其中市本级增长7.5%；一般公共预算收入增长8%，其中市本级增长8%以上；城镇居民、农村居民人均可支配收入增长与经济增长同步；全要素生产率稳步提高，节能减排完成省下达的目标任务。

　　今年，我们将紧紧围绕推动高质量发展，做好六方面重点工作：

　　（一）持续深化供给侧结构性改革

　　坚持质量第一、效益优先，推动质量变革、效率变革、动力变革，加快新旧动能接续转换，进一步增强经济发展的质量优势。

　　大力去除低效供给。强化“亩均论英雄”“创新论英雄”导向，综合运用用能、用地、排污、信贷等差别化要素配置政策，倒逼企业转型升级，力争整治提升、关停淘汰“低散弱”和“四无”企业（作坊）6000家以上，处置“僵尸企业”20家以上。严格能源“双控”管理，完善高耗能项目“用能置换”机制，强化高耗能行业能效对标。加强不良资产处置和“两链”风险化解，严厉打击违法违规金融活动，确保银行类金融机构资产质量继续保持全省最优。

　　改造提升传统动能。细化落实《“六大”传统行业改造提升行动方案》，积极运用“互联网+”“标准化+”“机器人+”“工业设计+”等手段，助力传统产业向数字化、智能化、高端化、绿色化方向迈进。实施企业股改挂牌上市和并购重组“凤凰行动”二十条，力争新增股改企业200家、上市公司11家，推动重点骨干企业实现规模与效益“双倍增”，培育一批细分行业的“单打冠军”“隐形冠军”。大力弘扬工匠精神，全面开展质量提升行动，加快推进国家检验检测高技术服务业集聚区建设，新增“浙江制造”品牌培育企业70家，通过认证30家，争创“全国质量强市示范城市”。多措并举降低实体经济成本，特别是制度性交易成本，竭力为企业减轻负担、排忧解难，让企业轻装上阵、倾力发展。

　　培育壮大新动能。新动能催生新经济，新经济开启新未来。让新经济尽快挑起大梁，对嘉兴转变发展方式至关重要。我们必须抢抓机遇、抢占风口，让新经济跑出又好又快的“嘉速度”。聚焦发展数字经济，加快新型智慧城市标杆市建设，推动互联网、大数据、人工智能与实体经济深度融合，在柔性电子、虚拟视效、机器人、无人机、氢动能等前沿技术领域大力培育多元化新增长点。全方位对接军队高端科技创新资源和成果，积极构建军民融合协同创新体系，大力推动“民参军”，主动承接“军转民”，加快长三角军民融合创新小镇落地。壮大平台经济、分享经济、体验经济、创意经济等新业态，推进南湖基金小镇、市本级大物流平台等服务业集聚区建设，抓住时机大力发展以红色旅游为牵引的全域旅游，力争服务业增加值增长8.5%。发挥有效投资对提高供给体系质量的关键性作用，突出抓好“百年百项”工程、“十百计划”和省市县长工程，确保全市交通基础设施、高新技术产业、生态环境和公共设施、民间投资均增长11%。

　　实体经济稳，则经济大局安。实体经济既是供给侧结构性改革的重要领域，也是全市经济发展的着力点和基本盘。对实体经济，我们必须高看厚爱、真心呵护、倾力支持，一定要让搞实体经济的企业有信心、有保障、有希望、有力量。

　　（二）深入实施创新驱动发展战略

　　创新是引领发展的第一动力，是建设现代化经济体系的战略支撑。要坚持把创新摆在首要位置，推动以科技创新为核心的全面创新，不断增强经济创新力和竞争力。

　　激发科技创新活力。牢牢抓住充分调动人的能动性这一根本，完善创新激励政策，推动创新绩效评价从重过程向重结果转变，赋予创新团队和领军人才更大的人财物支配权和技术路线决策权。改革科技管理制度，改进财政扶持资金投入方式，聚焦支持重大科技项目、重点科技平台和创新型企业，健全主体多元、渠道方式多样的科技金融服务体系。深化“泛孵化器”建设，深入实施科技企业孵化器倍增计划和高新技术企业“育苗造林”工程，促进一批综合型孵化机构向专业型方向转变，力争全市孵化机构数量达到100家以上、孵化面积800万平方米，新增高新技术企业200家、科技型中小企业500家。

　　打通融通创新通道。以“政产学研金介用”七位一体协同发展、融通创新为核心，大力推进科技成果产业化、本地化。加快建设各类创新平台，设立产业创新服务综合体培育基金，健全梯度培育机制，新增省级产业创新服务综合体4家，力争实现省级高新技术产业园区县（市、区）全覆盖。高水平建设科技企业研发机构，加快实现规上工业企业技术（研发）机构和亿元以上工业企业技术（研发）中心全覆盖。丰富省校合作内涵，创新运用“一院一园一基金一政策”模式，进一步提升浙江清华长三角研究院、浙江中科院应用技术研究院等新型创新载体与地方经济发展的融合度。协调开展沪嘉杭G60科创走廊三方规划编制工作，拓展科技创新券跨区域试点应用，推动三地共同发布科技创新政策、共同开展知识产权联合执法、共同组织专项推介活动，加快形成各类创新资源一体贯通、高效配置、开放共享的有利格局。

　　加大引才聚才力度。立足打造人才生态最优市，深化人才发展机制改革，大力实施“585”人才工程，进一步提升“创新嘉兴·精英引领计划”“星耀南湖·精英峰会”等品牌活动的海内外影响力，健全“海外工程师”“海纳孵化器”等柔性引才机制，全面落实人才新政，力争引进“千人计划”“万人计划”专家50名以上。加大对本土人才、高技能人才、基础性人才的培养力度。不唯地域、不求所有、不拘一格，以强烈的人才服务意识，营造爱才惜才、宜业宜居的人才发展环境，使各类人才人尽其才、才尽其用、用有所成。

　　发展依靠创新，创新唯靠人才。我们要视人才为第一战略资源，聚天下英才而成就之。使在嘉禾大地上有更多的“千里马”竞相奔驰，千帆竞发、百舸争流，汇聚起磅礴的创新伟力，助我嘉兴展翅高飞。

　　（三）更高水平推进改革开放

　　坚定“改革开放再出发”，协同推进深层次改革、全方位开放，为经济社会发展注入强大动力和活力。

　　全力深化“最多跑一次”改革。坚持效果导向，一切从方便群众和企业办事出发，深化“一窗受理、集成服务”，全面推行“无差别全科受理”，所有民生事项和企业办事事项实现“一次办结”，其中80%以上开通网上办理，50%以上的民生事项实现“一证通办”。深入开展“减证便民”行动，推广运用“刷脸通办”。调整完善公共数据和电子政务管理体制，加快推进数据共享交换，持续合力打破“信息孤岛”。实施企业投资项目承诺制改革，推行“标准地”制度，力争实现企业投资项目开工前审批全流程最多跑一次、最多100天。提升商事登记便利化水平，开展“证照分离”改革试点，统筹实施“多证合一、证照联办”。

　　加快推动重点领域改革。深化财税体制改革，完善全口径预算体系，全面实施绩效管理。创新政府产业基金引导方式，拓宽直接融资渠道，鼓励浙江南湖金融资产交易中心等新型金融市场主体创新发展。加快国有资产管理体制和国有资本授权经营体制改革，推进国有企业优化重组和混合所有制改革，推动嘉兴银行更好更快发展。建立重大基础设施资金统筹保障机制，深化PPP模式推广应用，探索开展存量资产转化为PPP试点。积极构建亲清新型政商关系，健全企业家参与涉企政策制定机制，激发和保护企业家精神，让企业家在市场竞争中有公平感、在合法经营中有安全感、在社会生活中有尊严感。

　　更高层次接轨上海。把接轨大上海、融入长三角作为落实省大湾区大花园大通道大都市区建设的主要载体，突出规划先行，坚持全域统筹“一盘棋”，坚持有所为、有所不为，聚焦交通、产业、公共服务等重点领域，全速推进省全面接轨上海示范区建设。编制沪嘉综合交通对接规划，制定三年行动计划，系统梳理一批铁路、轨道交通、公路、航道等重大项目，加快沪乍杭、沪嘉甬、通苏嘉等铁路项目前期工作。支持毗邻区域机制创新、先行先试，谋划建设临沪产业协同发展示范带。继续引进实施一批优质合作办学、办医项目，深化“一卡通”双向应用，不断提升示范区建设的影响力、承载力、竞争力。

　　加速形成对外开放新优势。握指成拳，打破以往各类产业平台“划地为界”“各自为政”的管理体制束缚，整合提升一批集聚高端外资的主平台，以大平台招引大项目好项目，力争引进世界500强、全球行业领先企业和总投资超亿美元项目30个以上。巩固贸易强市地位，实施“外贸小微企业三年成长培育计划”，积极应对国际贸易摩擦，在稳定主要出口市场份额的同时，引导企业主动参与“一带一路”建设，不断开拓新兴市场；大力发展跨境电子商务、市场采购贸易、外贸综合服务平台等新业态、新模式，促进外贸“优进优出”。

　　（四）着力构建城乡协调发展新格局

　　坚持统筹均衡，推动城乡一体化发展继续走在前列。

　　高质量推进新型城镇化。构建主体功能明确、区块定位清晰、特色差异鲜明、资源优化配置的全域统筹大格局，建成运行“多规合一”大数据平台，强化全域规划管控。严格落实土地利用总体规划，牢牢守住生态保护、永久基本农田保护、城镇开发边界“三条红线”。加快实施一批事关全域发展的重大工程，推进杭州湾跨海大桥北接线二期、杭平申线等项目建设，规划建设嘉兴高铁南站综合交通枢纽，做好苏台高速等项目前期工作，力争开工建设嘉兴军民合用机场。以迎接建党百年为契机，着力提升中心城市建设品质，加快南湖湖滨区域改造提升、子城城市客厅、市政务服务中心、市区快速路环线等“十大标志”工程建设，推动形象出新、功能优化。全面完成海绵城市试点任务，确保通过国家验收。深化交通治堵，实施秀洲大道延伸、亚太路改造等项目，打通纺工路北延、槜李路等断头路，畅通城市内循环。联动推进小城市培育试点、特色小镇创建、小城镇环境综合整治，进一步增强镇一级就近集聚人口、吸纳就业、促进城乡一体化发展的重要节点功能。

　　大力实施乡村振兴战略。加强调查研究，科学编制乡村振兴战略实施规划，启动创建十个乡村振兴示范镇、百个示范村、千个示范点。深化农业供给侧结构性改革，坚持质量强农、绿色兴农，高水平建设农业产业集聚区、特色农业强镇和田园综合体，加快构建现代农业产业体系、生产体系、经营体系。巩固和完善农村基本经营制度，统筹推进全域土地综合整治和土地承包经营权流转，发展以家庭农场为主要模式的适度规模经营，积极培育“农创客”“新农人”等新型职业农民，组建农业领创人才联盟，促进小农户与现代农业发展有机衔接。壮大村级集体经济，实施新一轮“强村计划”，确保年经常性收入100万元以上村达到80%以上。开展农村人居环境整治三年行动，争创省级美丽乡村示范镇7个，新增3A级景区村庄10个。完善乡村治理，塑造文明乡风、良好家风、淳朴民风。

　　健全城乡融合体制机制。统筹城乡规划布局，加快建立城乡统一的人才、土地、科技、资本等要素市场，引导和推动更多的要素向农业农村流动，促进城乡共同繁荣。探索宅基地所有权、资格权、使用权“三权分置”，多样化开展闲置宅基地和农房的综合利用。深化农村集体产权制度改革，全面实施农村集体资产清产核资，鼓励通过农业主体交叉持股、农民土地入股等形式，拓宽农民持股渠道，增加农民财产性收入。深化城乡基本公共服务均等化改革，推进城镇基础设施向农村延伸，高标准建设“四好农村路”，改造提升农村公路145公里。加快农村电网改造升级。完善农业转移人口市民化政策。

　　“农，天下之大本。”任何时候都不能轻视农民、忽视农业、漠视农村。我们一定要坚定不移，久久为功，想方设法让农业成为有奔头的产业，让农民成为有吸引力的职业，让农村成为安居乐业的美丽家园。

　　（五）持之以恒推进“美丽嘉兴”建设

　　牢固树立“绿水青山就是金山银山”的理念，全力打造人与自然和谐共生的江南水乡典范。

　　不断深化“三五共治”。围绕“五水共治”，严格落实“河长制”“湖长制”，统筹推进污水处理设施提升、水源地规范化建设、水系连通等工程，深化小微水体和近海海域污染整治，开展“污水零直排区”创建，力争Ⅲ类以上水质断面比重达到40%以上。围绕“五气共治”，打好蓝天保卫战，加大重点区域废气整治力度，持续做好烟花爆竹“双禁”和秸秆综合利用等工作，力争全市空气质量优良率达到80%以上。围绕“五废共治”，完善生活垃圾分类投放、收集、运输和处置体系，加快23个固体废弃物处置项目建设，确保年底实现全市危废处理能力基本自足。加大土壤污染治理力度，科学开展土壤污染防治工作。

　　推进低碳城市建设。倡导“低碳+产业”“低碳+城镇建设”“低碳+生活消费”，开展国家低碳城市试点工作。强化全社会用能管理，提高资源节约集约利用效率，实施100个绿色低碳改造项目，创建一批绿色园区、绿色工厂。大力发展光伏、光热、核电关联等产业，做大清洁能源产业基础，力争新增光伏并网容量200兆瓦，核电关联产业规模突破100亿元。进一步拓展平原绿化空间，规划建设一批美丽经济交通走廊、水乡湿地公园，加快构建“千里绿道网”，争创国家森林城市。

　　强化制度刚性约束。严格落实主体功能区规划和环境功能区规划，加强生态环境空间分区分类管理。完善排污许可制度，分行业规范发放排污许可证，逐步实现对固定污染源的全过程监管。保持环境执法高压态势，有序推进环保监测监察执法机构垂直改革，健全“黑名单”、信用评价、信息强制性披露等制度，严厉打击环境污染违法行为。加快中央环保督察和环境审计问题整改落实，全面建立生态环境状况报告制度，严肃生态环境损害责任追究。

　　喝上干净的水，呼吸新鲜的空气，头顶一片蓝天，随处有潺潺清水相伴，这是每一位市民的基本权利，更是政府的不懈追求。对污染防治、环境治理的攻坚战，我们必须坚决打赢到底，不获全胜，就决不收兵。

　　（六）切实保障和改善民生

　　紧扣群众关切，尽力而为、量力而行，一件跟着一件办，一年接着一年干，使嘉兴百姓生活更加幸福、更加安康。

　　提高就业和社会保障水平。优化就业创业服务，促进高校毕业生、残疾人、就业困难人员、农村转移劳动力等重点人群就业创业，切实做好退役军人安置工作。完善社会保障体系，规范各类社会保险制度衔接和待遇调整工作，建立统一的社会保障公共服务平台。构建“大救助”格局，健全政府救助、社会补充、群众互助的多元救助体系，加强对困难群众、特殊群体的兜底保障，实现精准帮扶、分类保障、应保尽保，着力打造更有温度的善治之城。坚持“房子是用来住的、不是用来炒的”定位，加快建立多主体供给、多渠道保障、租购并举的住房制度，启动建设棚户区安置住房1.6万套，建成保障性安居工程1万套以上。

　　发展公平而有质量的教育。改革学前教育管理体制，新增二级以上幼儿园15所，进一步提高公益普惠幼儿园的比例。促进义务教育城乡一体化发展，深化集团化办学和学校共同体建设，推动优质教育资源下沉，提升镇村教育品质。加快推进市中小学社会综合实践基地项目选址规划、清华大学附中实验学校落地、北师大嘉兴附中迁建等工作。坚持普通高中多元特色发展。密切产教融合、校地合作，完善现代职业教育体系，支持在禾高校发展。继续大力办好民办教育、特殊教育、继续教育和网络教育。扎实做好校园安全工作。

　　加快“健康嘉兴”建设。坚持“六医”统筹，加强与知名医学院校和高端医院的合作，培育名医名科名院。完善“双下沉、两提升”长效机制，提高家庭医生签约服务质量，健全基层医疗服务体系。实施智慧医疗惠民计划，全面建成人口健康信息平台，推广诊间结算、“云胶片”等方式，方便群众就医。推进市中医院改扩建、市医疗急救血液管理中心等项目建设，启动市第二医院迁建前期工作。全面推行长期护理保险制度，提高居家养老服务品质，实现全市养老机构、居家养老照料中心“医养结合”全覆盖。加强食品药品安全监督管理，让群众买得放心、吃得安心。加快市全民体育健身中心、中央体育公园等项目建设，推动全民健身和全民健康深度融合。办好世界女子大金属球锦标赛。

　　身心健康关乎千家万户、关系全面小康，更是社会主义现代化强市建设的应有之义。人民对美好生活的向往就是我们的奋斗目标，我们当以百倍的努力和不懈的行动，使“健康嘉兴”成为我们这座名城引以为豪的城市印记。

　　丰富群众精神文化生活。结合时代特点大力弘扬“红船精神”，深入践行社会主义核心价值观，高水平、全域化推进全国文明城市创建。深度挖掘嘉兴红色文化、运河文化资源，推出一批文艺精品力作，向建党一百周年献礼。扎实开展国家公共文化服务体系示范区后续建设，着力提升文化馆、图书馆总分馆和镇（街道）综合文化站服务效能，新增农村文化礼堂120家，努力使群众文化生活就近就便、多姿多彩。

　　推动社会治理共建共治共享。深化“自治、法治、德治”基层治理工作，提升“基层治理四平台”功能，打造社会治理的“嘉兴样板”。做好新居民工作。健全公共法律服务体系，落实普法责任制。创新信访工作方式，依法及时解决群众合理诉求。完善应急体系，全面构建重点风险隐患防控清单管理制度，提高防灾减灾救灾能力。严格落实安全生产责任制，坚决遏制重特大事故。推进平安嘉兴建设，依法开展扫黑除恶专项斗争，严密防范和坚决打击各类违法犯罪活动，全力打造中国最具安全感城市。支持工会、共青团、妇联、科协等群团组织改革发展，提升民族宗教、外事侨务、港澳台事务等工作水平。支持驻嘉部队建设，做好国防动员、国防教育、民兵预备役、人民防空、国家安全等工作，成功再创省级双拥模范城。

　　今年我们将继续按照民生实事“群众提、委员议、代表定、政府办”的理念，认真办理十件民生实事，解决好人民群众普遍关心的突出问题。

　　切实加强政府自身建设

　　新时代开启新征程，新征程呼唤新作为。我们将切实加强政府自身建设，加快推进政府数字化转型，推动政府治理体系和治理能力现代化建设，以拼搏奋进、砥砺奋斗的扎实业绩，回报全市人民的信任和重托。

　　坚持党的领导。把党的领导作为最高政治原则，提高政府工作的政治站位。牢固树立“四个意识”，增强“四个自信”，坚决维护以习近平同志为核心的党中央权威和集中统一领导，坚决贯彻党的基本理论、基本路线、基本方略，坚决落实中央大政方针和省委、省政府及市委决策部署。

　　推进依法行政。立治有体，施治有序。严格遵守宪法法律，丰富和完善“学法进行时”，切实提高政府工作人员尊法学法守法用法的意识，把政府活动全面纳入法治轨道。自觉接受人大依法监督、政协民主监督和监察监督、司法监督，加强政府内部层级监督和审计监督，主动接受社会和舆论监督。健全规范决策咨询、专家论证、公示听证等程序，提高决策科学化、民主化、法治化水平。全面深化政务公开，做好信息发布和政策解读。深入推进综合行政执法改革，深化“双随机、一公开”。按要求组织实施政府机构改革。

　　强化务实担当。不驰于空想，不骛于虚声。积极营造崇尚实干、行动至上的浓厚氛围，完善激励约束、容错纠错机制，旗帜鲜明地为敢于担当、踏实做事的干部撑腰鼓劲，坚决整治工作不落实、尸位素餐、做事敷衍塞责的行为。开展“大学习大调研大抓落实”行动，想群众之所想、急群众之所急，真正把功夫下到察实情、出实招、办实事、求实效、实打实上。“世界上的事情都是干出来的，不干，半点马克思主义都没有。”我们就是要干字当头，实干巧干苦干，干出精气神，干出新业绩，干出嘉兴的新风貌。

　　加强廉洁自律。不忘初心，方得始终。认真开展“不忘初心、牢记使命”主题教育。强化“一岗双责”，层层压实管党治党政治责任。严格遵守中央八项规定实施细则和省、市36条办法，持之以恒正风肃纪，防止“四风”问题回潮复燃。坚持标本兼治，加大违纪违法案件查处力度，围绕权力运行关键环节，不断健全不敢腐、不能腐、不想腐的体制机制，像精心呵护山清水秀的自然生态一样，大力营造风清气正的政治生态。

　　各位代表、同志们！时代是出卷人,我们是答卷人,人民是阅卷人。当前，嘉兴正处在转型升级与创新发展的关键时期，面对新的目标任务，我们深感使命光荣、责任重大。让我们更加紧密地团结在以习近平同志为核心的党中央周围，在中共嘉兴市委的坚强领导下，锐意进取，扎实工作，为把嘉兴建设成为富强民主文明和谐美丽的社会主义现代化强市而努力奋斗!
